# The Queen and the Cold Weather

**Published:** 1891-10-31

**Authors:** 


### The Doctor's Armchair.

### THE QUEEN AND THE COLD WEATHER.

"Truth" hears that Sir William Jenner has been moved by the Queen's contempt for the cold weather to send to her a seasonable remonstrance. Her Majesty, it seems, is in the habit of driving out in an open carriage under all sorts of atmospheric conditions. Neither cold, nor rain, nor even snow keeps her indoors, or persuades her to the stuffy security of a closed carriage. This almost reckless daring has alarmed Sir William Jenner, who may possibly have had experience of some of the Queen's long drives about Balmoral in open carriages in the midst of raging snowstorms, and with a north or easterly wind cutting him to the marrow. *Truth* suggests, with respectful and even affectionate loyalty, that Her Majesty is getting a little too old for such dangerous risks. It reminds her and her advisers of the fate of the Duke of Kent. The Duke, who had hardly had an ache or a pain in his life, thought himself invulnerable to cold or damp. He took a walk over the hills near Sidmouth on a wet, chilly day. On his return, though his boots and stockings were soaked, he appears to have altogether despised the idea of changing them. He sat down as usual to read the papers and write some letters, got chilled, and was dead in three days.

Open carriages, even in winter and bad weather, have great advantages over closed ones, for those who are well and strong. It is probable, too, that the Queen is much better able to resist the cold than Sir William Jenner. She has had life-long advantages of a health kind over that venerable and distinguished physician. Her Majesty has almost lived out of doors, and she has, moreover, had the unspeakable advantage of constant change of residence, each residence being not only in a most salubrious spot, but occupied at the time of the year in which its weather has been most perfect. Sir William Jenner, on the other hand, has, during the greater part of his life, lived in a close and stuffy house in dull and foggy London. For many years in the earlier part of his career he, no doubt, worked painfully hard, and amid the pressure of a considerable variety of anxious worries and cares. He is probably, therefore, much less vigorous than the Queen, and has far less of the elasticity of "youth in advancing age." The Queen is still young, notwithstanding her three-score years and ten; and she evidently cannot bring herself to admit, even by implication, that Grey-haired Time is laying his hand upon her.

One cannot but sympathise with Her Majesty in her choice of an open carriage, even in the wild and wintry weather of Balmoral. In a close brougham or landau it is hardly possible to "take the air" at all. If the windows be shut the carriage becomes more stuffy than a drawing-room: if they be open the draught is more direct and dangerous than that felt in an open carriage. What one feels is that in winter the best way of taking the air is to walk; and instead of attempting too long an excursion at once, to be provided with outdoor garments which can be readily put on and taken off, and to go for three or four, or more walks a day, instead of one. If a mere change of scene and circumstance be needed on a very cold or wet day, then, undoubtedly, a brougham or some other close carriage should be ordered out. But even in winter, and on severely cold days, if the weather be neither wet nor very windy, an open carriage, with proper precautions, is the best for people in good health. What are those precautions? The carriage should be deep and roomy, well-cushioned, and well-jointed, and should, by preference, be a landau with the front elevated for a screen to keep off the direct rush of the air. Foot-warmers should always be used, and thick wraps and rugs. A sealskin or other fur coat or mantle should be worn, with a well-fitting and warm hat or bonnet; and in the case of a lady, a thick, soft, Shetland veil. Moreover, care should be taken to avoid driving when hungry; and, indeed, it is wise always to take some hot and comfortable food just before starting. Thus clothed, warmed, and defended, within and without, even aged persons, of good health, who have been accustomed to living in the open air, may generally indulge in open-carriage exercise, on suitable days, all the year round.

But it is not to be supposed that Sir William Jenner's advice to the Queen is to be disregarded either by Her Majesty or any other person. Advancing years make their claims upon all of us, and those claims cannot be evaded. The heart will become a little more feeble in spite of all that rank and wealth can do to maintain its vigour. The blood will turn thinner and poorer, and the vessels that carry it to and from the tissues less vigorous and elastic in their propulsive energy. All the powers of resistance to cold and other adverse circumstances will gradually, but inevitably, diminish. Age will creep on, alike in Queen and subject.

In growing age, discretion is eminently the better part of valour. Colds, catarrhs, chills of the liver, and even chills of the skin are to be avoided like watchful enemies in an invaded country. They are not to be nervously feared and run away from, but to be dealt with intelligently and courageously; always with the proper comprehension of their danger, but also with the instructed consciousness that they can be avoided by proper means.

The Queen is a living and comforting object lesson to all people who are beginning to be aged. Mr. Gladstone is another. To be well and happy in age requires the exercise of a good deal of intelligence and resolution. Age may be happy and healthy too; let us thank God for that. But it requires a good deal of docility on the part of aged persons themselves, and abundant sympathy and tender watchfulness on the part of those who constitute the little world of the aged, to ensure their happiness and health. A fellow feeling has, no doubt, prompted Sir William Jenner to speak to the Queen on the subject of open drives in cold and rainy weather. It is a fellow-feeling alone which can help any of us to minister with skill and intelligence to those who are much older than ourselves. We have to remember that though we may not be old now, we shall certainly be old in due time, unless we die prematurely. Sir William Jenner's letter cannot but be of great service, not only to the Queen herself, but also to all aged persons, and to their relations, friends, and servants, who care for and minister to them. Of all debts which the world is called upon to pay, that which it should always discharge most willingly is the debt of gratitude to the aged who have served it faithfully, well, and long.

